# Bee-safe peptidomimetic acaricides achieved by comparative genomics

**DOI:** 10.1038/s41598-022-20110-0

**Published:** 2022-10-14

**Authors:** Vikas Jindal, Daqi Li, Leslie C. Rault, Soheila Fatehi, Rupinder Singh, Moritz Mating, Ye Zou, Ho-Leung Ng, Krzysztof Kaczmarek, Janusz Zabrocki, Shunhua Gui, Guy Smagghe, Troy D. Anderson, Ronald J. Nachman, Yoonseong Park

**Affiliations:** 1 Department of Entomology, Kansas State University, 123 Waters Hall, Manhattan, KS66506 USA; 2 Department of Entomology, Punjab Agricultural University, Ludhiana, 141004 India; 3 Institute of Plant Protection, Shanxi Academy of Agricultural Sciences, 81 Longcheng Street, Taiyuan, 030031 Shanxi China; 4 Department of Entomology, University of Nebraska, 103 Entomology Hall, Lincoln, NE 68583 USA; 5 Department of Biochemistry and Molecular Biophysics, Kansas State University, Manhattan, KS66506 USA; 6 Insect Neuropeptide Laboratory, Southern Plains Agricultural Research Center, U.S. Department of Agriculture, 2881 F&B Road, College Station, TX 77845 USA; 7 Institute of Organic Chemistry, Lodz University of Technology, 90-9024 Lodz, Poland; 8 Lab Agrozoology, Department Plants and Crops, Ghent University, Coupure Links 653, 9000 Ghent, Belgium

**Keywords:** Toxicology, Target identification

## Abstract

The devastating *Varroa* mite (*Varroa destructor* Anderson and Trueman) is an obligatory ectoparasite of the honey bee, contributing to significant colony losses in North America and throughout the world. The limited number of conventional acaricides to reduce *Varroa* mites and prevent disease in honey bee colonies is challenged with wide-spread resistance and low target-site selectivity. Here, we propose a biorational approach using comparative genomics for the development of honey bee-safe and selective acaricides targeting the *Varroa* mite-specific neuropeptidergic system regulated by proctolin, which is lacking in the honey bee. Proctolin is a highly conserved pentapeptide RYLPT (Arg-Tyr-Leu-Pro-Thr) known to act through a G protein-coupled receptor to elicit myotropic activity in arthropod species. A total of 33 different peptidomimetic and peptide variants were tested on the *Varroa* mite proctolin receptor. Ligand docking model and mutagenesis studies revealed the importance of the core aromatic residue Tyr2 in the proctolin ligand. Peptidomimetics were observed to have significant oral toxicity leading to the paralysis and death of *Varroa* mites, while there were no negative effects observed for honey bees. We have demonstrated that a taxon-specific physiological target identified by advanced genomics information offers an opportunity to develop *Varroa* mite-selective acaricides, hence, expedited translational processes.

## Introduction

The *Varroa* mite (*Varroa destructor* Anderson and Trueman) is an obligatory ectoparasite of honey bees. Host expansion of the *Varroa* mite from the Eastern honey bee *Apis cerana* to the Western honey bee *A. mellifera* is associated with the worldwide dispersion of the mite in the last century^1^ and has caused a major threat to the maintenance of healthy bee colonies^2^. Infestation by *Varroa* mites causes direct damage to the bee colony and facilitates the transmission of pathogens^3,4^. Control of the *Varroa* mite in beehives has relied heavily upon the use of synthetic and natural acaricides^5^.

The use of chemical acaricides in beehives to control *Varroa* mites requires stringent criteria. Finding selective acaricides that are nontoxic to honey bees is a challenging task because the arachnids and insects belong to the same phylum, Arthropoda, and diverged approximately 750 million years ago^6^. In addition, acaricides and their metabolized forms need to be without adverse effects on human health because of the consumption of the products in apiculture. Moreover, resistance to acaricides in the mites became another huddle in the control of the *Varroa* mites^7–10^. While new acaricidal compounds have been developed that show improved selectivity, new approaches have also been sought by taking advantage of the available genome sequences of the *Varroa* mite and honey bee and of new biotechnologies. For instance, RNA interference (RNAi) targeting the gene(s) specific to the *Varroa* mite has been tested for efficacy in apiculture^11^. The RNAi strategy was combined with the engineered symbiont-mediated delivery of double stranded RNA^12^. We have also proposed a new approach by targeting a *Varroa* mite-specific signaling system that is absent in honey bees^13^.

Our recent efforts included the development of highly selective acaricidal compounds using peptidomimetics targeting the neuropeptidergic system^13^. The rationale for this approach is based on comparative genomics between the *Varroa* mite and honey bee. A peptide signaling system that is present in *Varroa* mites but not in the honey bee genome could be an ideal target for this strategy. Previously, our study of peptidomimetics focused on two closely related neuropeptidergic systems^13^, namely, tachykinin-related peptide (TRP) and natalisin (NTL), where honey bees lack NTL and the NTL receptor. The study found that peptides carrying the C-terminal motif FWxxRamide are highly specific to interaction with the *Varroa* TRP receptor but not to the honey bee TRP receptor.

In this study, we applied a similar principle to the proctolin neuropeptidergic system, but without the complexity of two closely related peptidergic systems in NTL and TRP. Proctolin is a pentapeptide with the conserved sequence RYLPT (Arg-Tyr-Leu-Pro-Thr) and has been reported to elicit myotropic activity in many different arthropods. This peptide has thus far been found only in arthropods based on homologous sequences, and is absent in vertebrates. Interestingly, this peptide and the receptor have also not been found in Lepidoptera and Hymenoptera, including the honey bee, providing a highly selective acaricidal target. We tested a functionally expressed *Varroa* mite proctolin receptor against a series of proctolin analogs as well as peptidomimetics containing biostability enhancing components. In addition, we tested the proctolin mimetics, showing high activities on the receptor, for the oral toxicities on the *Varroa* mites. The current results show that proctolin peptidomimetics could represent a highly promising approach for the development of bee-safe acaricidal agents for the management of the *Varroa* mite.

## Results and discussion

### Arthropod proctolin and the G protein-coupled receptor

**Figure 1:**
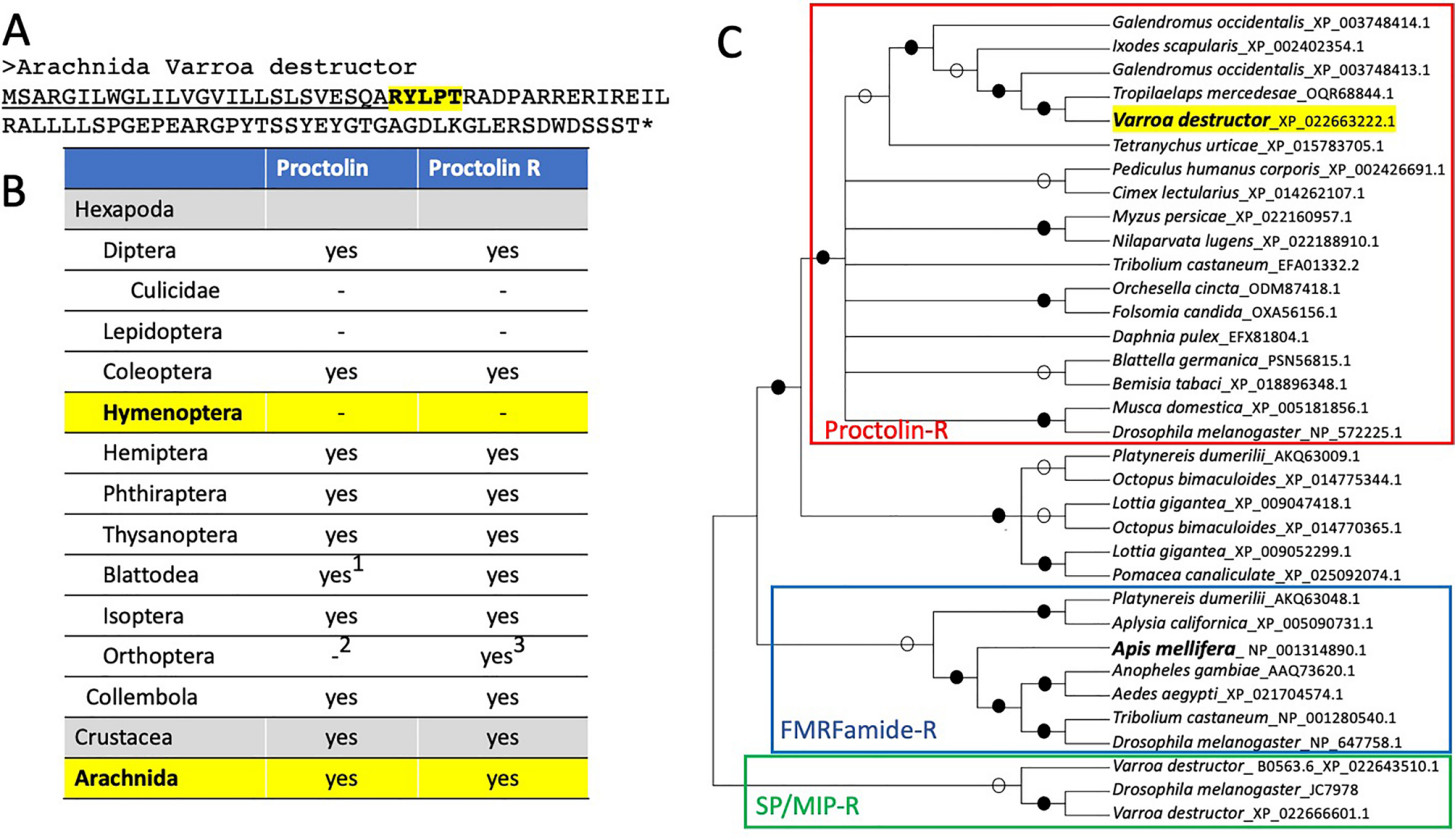
Taxonomic distribution of proctolin signaling system. The sequence of the proctolin of *Varroa desructor* (**A**), distribution of proctolin orthologous genes in arthropods (**B**) and phylogeny of the proctolin receptors rooted by FMRFamide receptors and sex peptide/myoinhibitory peptide receptors (**C**). The footnotes 1, 2, and 3, for Blattodea and Orthoptera proctolin is described in Supplementary data 1. Highlighted raw in B is for the taxonomic groups that is relevant to this study. Solid and empty circles in C are for bootstrapping values > 85% and > 50%, respectively.

Proctolin and its receptors are widely distributed in arthropods, including insects, crustaceans, and arachnids (Fig. 1A–C, Supplementary Information). The *Varroa* mite gene encoding proctolin has a short open reading frame with a predicted N-terminal signal peptide. Immediately after the canonical signal peptide, the proctolin sequence followed and ended with a single R, a presumed monobasic cleavage site (Fig. 1A). In a survey of genes encoding proctolin in other taxa, almost all proctolin sequences identified in our search contained a strictly conserved pentapeptide followed by an R for monobasic cleavage with few exceptions.

The human louse (*Pediculus humanus*) contains the sequence RWLPT, where the second aromatic amino acid W2 replaced the aromatic Y2 in the consensus sequence. The rhinoceros beetle (*Oryctes borbonicus*) has RYLPA featuring a replacement of T5 with A5^14^. Lady beetles (*Coccinellidae*) encode for RYLST, replacing P4 with S4^14^. A previous study reported a RALPT variant (replacing Y2 with A2) in addition to the presence of the typical RYLPT in Colorado potato beetle (*Leptinotarsa decemlineata*)^15^. However, a search of the Colorado potato beetle genome^16^ identified only RYLPT, and the Y2 to A2 replacement was not identified in the Blast search algorithm (PAM30, E-value threshold: 100). Overall, natural variations of proctolin were found, but with a limited number of cases involving the 4th or 5th amino acid residues, while W2 replacement retains the aromatic side chain in the tryptophan.

A proctolin receptor was first identified in *D. melanogaster*^17,18^ while the peptide proctolin was first described in the American cockroach in 1975^19^. In a phylogenetic analysis and in Blast searches for the proctolin receptor, an FMRFamide receptor was found to be the closest to the proctolin receptor group. The sex peptide receptor (also known as the myoinhibitory peptide or allatostatin B receptor) was the next closest group of G protein-coupled receptors (GPCRs, Fig. 1B). Interestingly, analysis of the basal lineages of Bilateria, lophotrochozoans including mollusks and annelids, revealed that a group of GPCRs were closely related to and grouped with the proctolin receptor of arthropods. Proctolin-like sequences in these taxa were not found in our blast searches. The authentic ligand for this group of receptors is expected to be similar to proctolin and remains to be uncovered.

In a survey of genome sequences, the *proctolin* gene was not identified in the insect orders Lepidoptera and Hymenoptera or the family Culicidae of the order Diptera, which includes mosquito species, important vectors of disease in human and animals. These species also lacked proctolin receptor orthologues. Based on the punctuated groups of taxa lacking proctolin and its receptor and the availability of many genome sequences for the species in these groups of insects, it is likely that they truly lack both proctolin and the receptors in independent evolutionary lineages. The translated sequences identified in the search are in Supplementary material 2.

In the case of Blattodea, where the first proctolin was isolated^19^ (Supporting information.1), the proctolin sequence was identified only in the genome sequence of *Zootermopsis nevadensis* (Blattodea; Isoptera). And, only a part of the prepropeptide encoding C-terminal amino acids lacking the N-terminal part of the mature proctolin sequence was identified in *Periplaneta americana*, *Blattella germanica*, and *Cryptotermes secundus* (Supplementary data). Proctolin receptors were also found in *Periplaneta americana*, (PGRX01000220.1: 583,757.0.612274) and in *C. secundus* (Blattodea; Isoptera, NEVH01012087.1: 3,622,722.0.3628892). The searches in the multiple species in Orthoptera genomes found similar results. The proctolin sequence was not identified in the genome sequences of Orthoptera, although proctolin was extensively studied in locust^20–24^. Only a partial sequence covering the receptor ortholog was identified in *Laupala kohalensis* (NNCF01130566.1:-:666,828.0.614799) and *Locusta migratoria* (AVCP010976312.1:2376.0.2423). The identification of only partial sequences of *proctolin* genes and its receptor in Blattodea and Orthoptera is likely due to incomplete genome sequences and annotations, suggesting that these taxa likely retain the proctolin signaling pathway.

A number of previous reports contradicted what we found in the genome sequence survey, i.e., the lack of proctolin system in the Orders Lepidoptera and Hymenoptera. Proctolin immunoreactivity has been described in the gypsy moth^25^ and *Manduca sexta*^26^. A chromatographic isolation followed by cockroach hindgut bioassay described extremely low quantities of proctolin in honey bee and tent moth (*Malacosoma americana*): 0.12 and 0.17 mg/kg of tissue, respectively^27^. Proctolin activity on the *Bombyx mori* larval gut has been previously found, though an extremely high concentration of 180 µM is required for hindgut contraction, and 10 µM is required in the midgut to slightly reduce leucine uptake^28^. In the honey bee, an extremely high dose of proctolin delivered via injection, 1 µL containing 1 µg (1.54 mmoles), increased the egg-laying activity of the queen^29^. However, in these studies, the bioactivity was very weak with extremely high concentrations of proctolin required for the bioacivities. The bioactivities described in these studies are unlikely a consequence of the natural endogenous bioactivities of proctolin.

### Proctolin-receptor docking model

**Figure 2:**
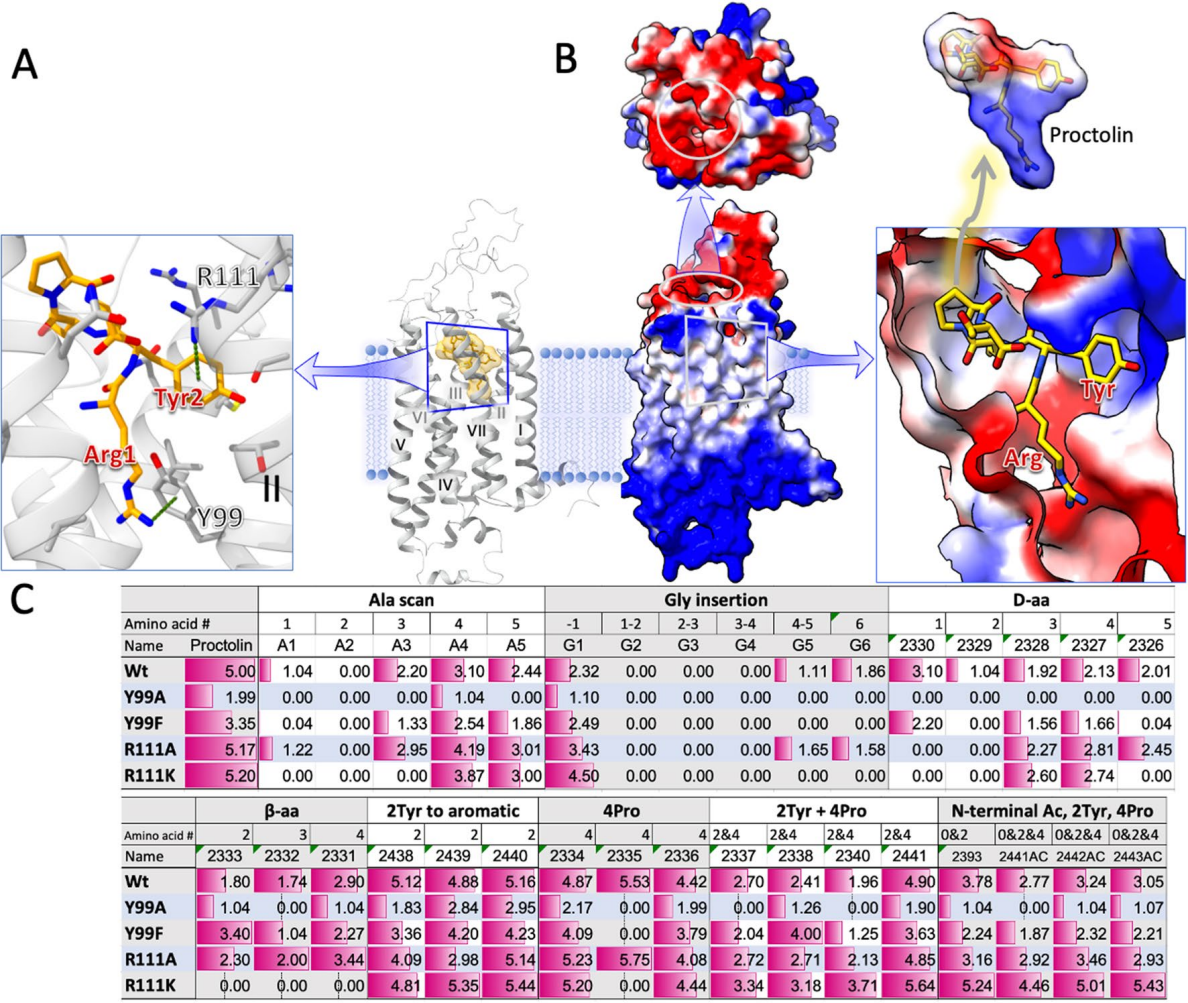
In silico docking model. The importance of cation-π interactions of Arg1 and Tyr2 of the proctolin (**A**), the charges of receptor and ligands on the docking model (**B**), and the mutated ligands and receptor activities tested on the receptors expressed in the CHO cell (**C**). In B, blue is for positive and the red is for negative charges. In C, the values are normalized values for the log10-based scale activities relative to the 5 as the standard for wild-type receptor with the proctolin.

To study the activities of proctolin peptidomimetics, we investigated the receptor by in silico docking modes and in vitro assays on the heterologously expressed *Varroa* mite proctolin receptor in Chinese Hamster Ovarian (CHO) cell that has been established for various insect GPCR expression^30,31^. The structure of proctolin receptor was built using the GPCR I-TASSER (Iterative Threading ASSEmbly Refinement) server^32–35^, and the docking with protcolin was simulated by an Induced Fit Docking (IFD) method^36^. A QM-Polarized Ligand Docking method revealed the binding pocket of the proctolin receptor (Fig. 2). The docking model shows the N-terminal Arg1 and Tyr2 of the ligand interacted with the Tyr99 and Arg111, respectively, by forming cation-π interactions with the distances 3.9 and 3.6 Å (Fig. 2A). These first two residues of proctolin are found to be important for access to the binding site and anchoring to dock into the receptor pocket. Cation-π interactions, a strong noncovalent binding interaction, appear to play unique and important role for proctolin docking on the ligand binding pocket. We also found that the extracellular surface of the receptor and the binding pocket including the region accommodating the positively charged Arg1 of the proctolin are generally negatively charged (Fig. 2B).

The importance of cation-π interactions was further supported in the mutagenesis of the proctolin ligand and proctolin receptor. The changes in Tyr2 of proctolin to other natural and non-natural aromatic amino acids generally retained the activities on the receptor in general (Table 1 and Fig. 2C), whereas Tyr2 to Ala mutation almost abolished the activity of the ligand (Table 1). Interestingly, however, the R111A (Arg111 to Ala) and R111K mutations on the receptor, which is the predicted receptor residue interacting with Tyr2 of the ligand, caused only minor changes in the receptor activities to the proctolin mimetics. These results imply that the aromatic ring in the Tyr2 interacts not only with the binding pocket, but functions for accessibility to the binding site. The cation-π interactions predicted for Arg1 of proctolin with Y99 of the receptor is supported by the mutations of the receptor Y99A and Y99F. The Y99F-mutated receptor with the aromatic residue maintained the activities, whereas Y99A lost the activities to various proctolin and its mimetics. The roles of cation-π interactions in the ligand binding sites and the amino acid residues lining the active site gorge need to be further studied.

**Table 1:**
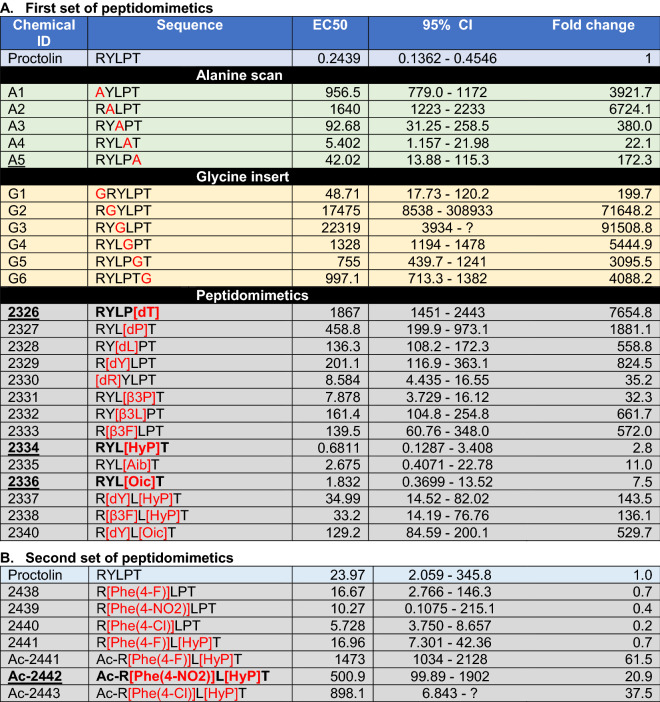
Activities of proctolin and its mimetics on the *Varroa* mite proctolin receptor. The modified amino acids are in red in the amino acid sequences. Bold and underlined peptidomimetics are the ones further studied in feeding assay. [d] is for D- amino acid, [β3] is for beta -3 amino acids, [Hyp] is for hydroxy proline, [Aib] is for 2-amino butyric acid, and [Oic] is for octahydroindole-2-carboxylic acid, and [Phe-4] is for modified phenylalanine at the 4 position. Note that the second set of peptidomimetics were tested on the cells selected for stable expressions of the receptor showing significantly lower activity to proctolin and to other analogues than those in the first set using the cells with transient expression of the receptor.

### Activities of proctolin analogs on the Varroa mite proctolin receptor

The receptor expressed in the heterologous expression system showed strong reactivity to proctolin, exhibiting an EC_50_ of 0.24 nM (Table 1). The negative control, transfection of the empty vector, did not show any activity at a high concentration of 10 µM proctolin. The receptor was also tested with a variety of peptide analogs and peptidomimetics.

**Figure 3:**
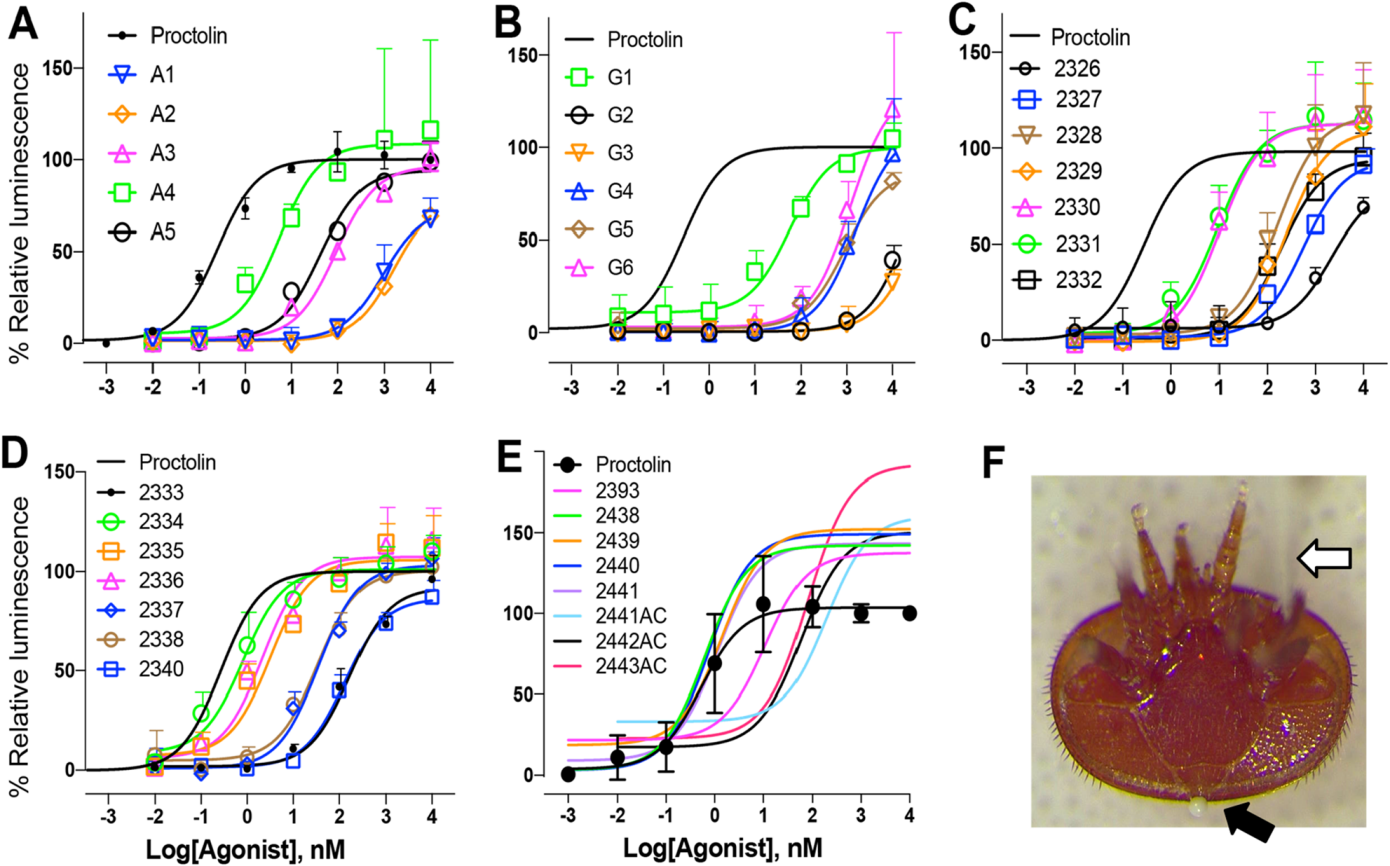
Activities of proctolin peptidomimetics. Log-dose response curves showing the activities of proctolin analogs and peptidomimetics on the *Varroa destructor* proctolin receptor (**A** to **E**), and *Varroa* mite showing excretion induced by injection of proctolin solution (**F**). In F, the white arrow is for the location of microinjection needle and the black arrow shows the excretion from the anus of *Varroa* mite.

An alanine scan series of analogs was evaluated to determine the importance of the side chains of specific amino acid residues (Table 1). The first two amino acids R1 and Y2 are the most important, while the fourth residue P4 can be replaced by A with only minor loss of the activity of the receptor. The replacement of the third and fifth amino acids (L3 and T5, respectively) to A resulted in moderately reduced activity. Therefore, the first two amino acids R and Y likely contain side chains that interact strongly with the receptor. We used glycine insertions to examine the importance of any two consecutive amino acid residues. The insertion of G at the position between R1 and Y2 and between Y2 and L3 dramatically reduced the activity (Table 1 and Fig. 3). All other G insertions resulted in significant reductions in activity except the N-terminus attachment of G, which resulted in a minor loss in activity (Table 1 and Fig. 3). The space in the N-terminus may allow chemical modification that offers increased permeability and blocks the aminopeptidase N mediated degradation^37^ of the proctolin peptidomimetics.

### Activities of proctolin peptidomimetics on the Varroa mite proctolin receptor

A series of peptidomimetic analogs of proctolin were chosen for synthesis and receptor evaluation that incorporated components that can enhance resistance to peptidase enzyme hydrolysis, i.e., biostability (including such enzymes as ACE, neprilysin, and aminopeptidases). The first set of five analogs involved sequential replacement of each of the amino acid residues with a D-amino acid. D-amino acids are not recognized by peptidase enzymes and can confer peptidase resistance to adjacent peptide bonds. Most D-amino acid replacements led to significant loss of potency, with the major exception being the replacement of the N-terminal R with dR in 2330, which retained activity at 8.6 nM for the EC_50_ (Table 1). This modification is of importance as it can protect the N-terminus from aminopeptidase degradation.

A set of analogs involved the replacement of amino acid residues with β-amino acids (Table 1); which are also not recognized by peptidases and can confer resistance to adjacent peptide bonds to enzyme hydrolysis. The amino acids chosen for replacement depended on the commercial availability of appropriate β-amino acids. Thus, L and P were replaced with β^3^L (2332) and β^3^P (2331), respectively. Finally, the aromatic Y residue was replaced with aromatic β^3^F (2333). Of these three analogs, analog 2331 (β^3^P for P) retained high potency with an EC_50_ of 7.9 nM.

The P (Pro) amino acid is an important residue for the stabilization of secondary structures such as β-turns, and this property was therefore retained in each of the members of the next series of analogs. Here, the P was replaced with either a modified, sterically-hindered proline analogs hydroxyproline (Hyp) and octahydroindole-2-carboxylic acid (Oic), or the sterically-hindered, turn-promoting residue aminoisobutyric acid (Aib). One analog in this set also incorporates an acetyl group (Ac) at the N-terminus to increase resistance to aminopeptidase attack. As observed with the high activity retained by analog 2331 (β^3^P for P), these P4 replacement analogs similarly demonstrated high potency retention. Analog 2334 (with Hyp) exhibited high potency with an EC_50_ of 0.68 nM, only 2.8 times less potent than the natural peptide. This analog also previously showed high activity in in vivo proctolin myotropic assays^38–45^. Analogs 2336 (with Oic) and 2335 (with Aib) also retained relatively strong potencies in the nanomolar range, with EC_50_’s of 1.8 and 2.7 nM, respectively. These sterically-hindered Pro replacements can confer greater biostability to the analogs that incorporate them^46,47^.

Additional analogs featured double-replacement analogs to potentially confer enhanced peptidase resistance to every peptide bond in the proctolin sequence. A component that enhances stability is adjacent to each peptide bond in the sequence of this analog pair. In this pair, sterically-hindered P replacements were coupled with either a replacement of Y with dY (2337 or 2340) or with β^3^F (2338). Despite the major modifications incorporated into analogs 2338 (R[β^3^F]L[Hyp]T) and 2337 (R[dY]L[Hyp]T), the two retained full efficacy (maximal response) and a relatively strong potency with EC_50_’s of 33 and 35 nM, respectively. Indeed, in each case, the potency of these double-replacement analogs at positions Y2 and P4 exceeded that of the corresponding analog (2333 and 2329, respectively) with only a single replacement at the Y2 position by a factor of 8 and 4, respectively. Analog 2340 (R[dY]L[Oic]T) was less potent than 2337 and 2338 by approximately an order of magnitude. Mimetic analogs featuring enhanced biostability have an advantage over native peptides in that they can exist and remain active for a longer period in the hemolymph. The analogs 2338 and 2337 may provide leads for biostable mimetic analogs that can disrupt the processes mediated by proctolin in parasitic mites.

The last set (the second set in Table 1) of peptidomimetic tested were the proctolin variants at the Y2 position with phenylalanine carrying the aromatic ring with 4-F, 4-NO2, 4-Cl, and with additional variations for [HyP] replacing at the 4th P and for Ac at the N-termini. This set of peptidomimetics showed high activities including hyper activities that have higher activities than endogenous proctolin, although the second set of ligands tested on the cells selected for stable expressions of the receptor showed significantly lower activity with two orders of magnitude to proctolin and to other analogues (Table 1 and Fig. 3). Replacement of the Y2 to [Phe(4-NO_2_)], [Phe(4-Cl)], [Phe(4-F)], [Phe(4-F)] showed even higher activities than proctolin. A double-replacement, R[Phe(4-F)]L[HyP]T, also had higher activity than proctolin, while it had slightly reduced activity than the single replacement R[Phe(4-F)]LPT (Table 1).

An interesting observation in this study is that the highly active ligands often display higher efficacy (maximal response) than proctolin at high concentrations (Fig. 3). A4, G6, 2330, 2331, 2334, and 2336 are in this category; the activities of these ligands at the 1 μM and 10 μM were higher than that of proctolin. Previous studies proposed that binding of the agonist and elicitation of conformational changes to initiate activation of down stream responses are sequentially distinctive processes in the β2 adrenoceptor^48–50^. The highly active ligands in this study may also have influenced receptor activation of the proctolin receptor in a similar fashion. Certain configurations of the ligand may elicit more efficient conformational changes in the receptor than the endogenous ligand, despite featuring lower binding kinetics.

The activities of numerous proctolin peptidomimetics have been previously tested in various biological systems^38–45^. The major systems used for assessing the bioactivities of peptidomimetics were assays for cardioacceleration, gut motility, and oviduct motility in cockroach, locust, and beetle species. The results of these studies allow an excellent comparison to the results of our study using the proctolin receptor to directly assess the activities of the peptidomimetics. We find a general consensus in comparisons between our peptidomimetics in *Varroa* mite receptor activity and earlier studies using assays on myotropic activities in other species of insects. In Table 1, the peptidomimetics with gray backgrounds were inactive, and the bold and underlined ones were active in the earlier bioassay systems. Generally, where comparisons can be made, the peptidomimetics with a < 50 nM EC_50_ were also active in one of the early bioassay systems, with the exception of the R1 replacement by dR, which had an EC_50_ of 5.62 nM, only 26 × lower than native peptide activity, but no activity in earlier in vivo bioassays. This exception and difference could be due to structural variations between the receptors in the different species.

A previous study has demonstrated that the replacement of Y2 with analogs of Y or F incorporating modifications at the aromatic ring led to highly active proctolin analogs, in some cases with even higher activities than that of endogenous proctolin in the bioassay systems. This suggests that future studies investigating similar modifications of the aromatic moiety at the Y2 position could lead to biostable mimetic proctolin analogs with even greater potency and efficacy on the *Varroa* mite receptor.

### Peptidomimetics toxicity to Varroa mites and to the honey bee

**Figure 4:**
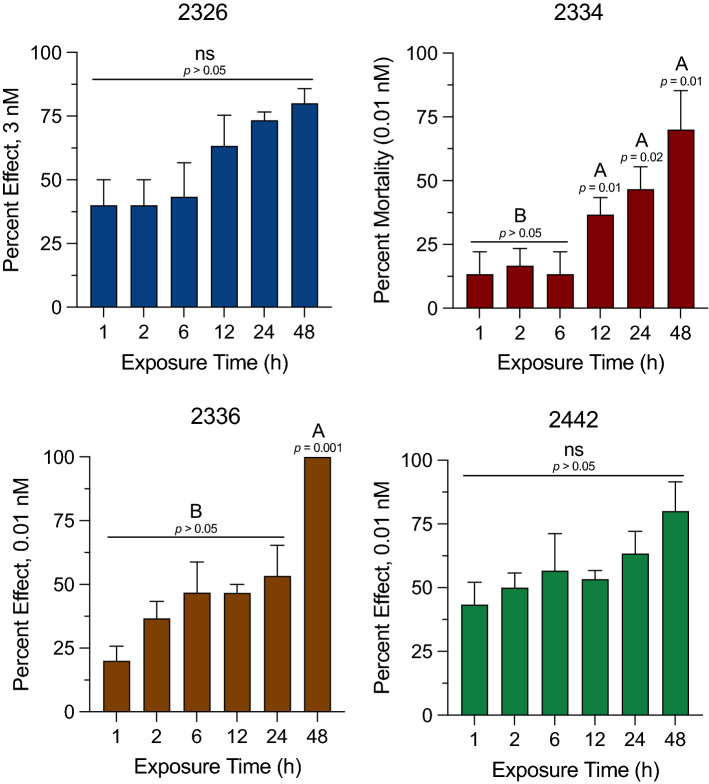
Proctolin peptidomimetics activities. Time-dependent efficacy of peptide mimics to *Varroa* mites. Immobilized Female *Varroa* mites were topically treated on the gnathosoma; 2326 at 3 nM and 2334, 2336, and 2442 at 0.01 nM and the number of mites affected (i.e., paralyzed or dead) was recorded for 1 to 48 h post-treatment. Vertical bars represent the mean ± standard error. Different letters above the bars indicate a significant difference between the mean percent effect at each time point using a one-way ANOVA with a Tukey’s post-hoc test (*p* < 0.05). The mortalities in water controls were ~  < 10% at 48 h exposure.

Four peptidomimetics tested for the bioassays were three strong agonists 2334, 2336, and Ac-2442, and an inactive agonist 2326 determined on the *Varroa* mite proctolin receptor. Initially, the highly active agonist 2334 on the receptor was directly injected into the body cavity (~ 5 nL of 200 µM) to test the direct biological activities. The injection immediately induced excretion of a drop of fecal material through the anus (3/4 individuals) while the H_2_O control did not cause excretion or other noticeable change (0/4, Chi-square test* p* < 0.05). This result indicates that the *Varroa* mite proctolin activity includes activation of excretory system which is a well-documented activity in other insects although the study needs to be further expanded. In the continued bioassay, the toxicities were assessed by counting paralysis/death in feeding assay. A drop of 200 nL solution was placed on the gnathosoma of an immobilized mite for forced feedings of active components. Low levels of acute toxicities of peptidomimetics were indicated by lower than 50% effects within 6 h after the treatments. The toxic effects were increased over time and reached 100% of the effects in the 48 h treatments of 2336 (Fig. 4, ANOVA * p* < 0.05). In H_2_O control, about 10% or less of the number of individuals were paralyzed/dead. Accurate cause of the paralysis/death remains yet to be studied.

Acute toxicity of the peptidomimetics on the worker honey bee was tested for 2439, 2334, 2336, Ac-2442, and H_2_O control by injections of the compounds into thorax. Injections of 50 nL solutions at the 200 µM concentrations did not show any negative effects in 24 h observations after the injections (n = 4 for each compound). The assay was expanded to study potentially a longer effect on the honey bee by 7 days of treatments for compounds 2330, 2331, 2334, 2335, and 2336. We found no statistically significant differences in sugar consumption rate and survival (Table 2).

**Table 2:** Percent survival of honey bee (*Apis mellifera*) and amount of sugar water uptake after injections of proctolin analogs. Sugar water consumption was measured at the day 3 and the survival was counted in the seventh day after treatment. The statistics were a Chi-square test for the survivorship and Student’s t-test for the sugar water consumption. No statistical difference compared to the water control was found at the *P* = 0.05.

Analogue	n	Survival (%)	Amount of sugar water uptaken per bee in 3 days (mean + SD, mg)
Water control	30	70	279 ± 65
Proctolin	30	73	278 ± 58
No-injection	30	77	272 ± 51
2334	30	73	281 ± 52
2331	30	70	271 ± 67
2336	30	70	283 ± 55
2335	30	60	273 ± 46
2330	30	63	289 ± 69

In addition, the activities of the proctolin peptidomimetics were tested on the honey bee FMRFamide receptor that is the most closely related to the proctolin receptor (Fig. 1). Honey bee FMRFamide receptor was cloned and expressed in the same system as described for the proctolin receptor. Proctolin and its peptidomimetics show no activities while the honey bee FMRFamides showed strong activities on the honey bee FMRFamide receptor.

## Conclusions

A new approach to developing selective acaricidal compounds targeting a *Varroa* mite specific neuropeptidergic system is outlined in this study. We have successfully developed an assay system using the *Varroa* mite proctolin receptor. Peptidomimetics were shown to retain relatively strong activity on the *Varroa* mite receptor and provide guidelines for the development of 2nd generation analogs of higher activity and bioavailability. In particular, two analogs that feature double replacements offer enhanced peptidase protection to all peptide bonds and retain full efficacy and significant retention of potency and represent promising lead analogs. The peptidomimetics pre-selected by the receptor assays showed significant activities in *Varroa* mite feeding assays at very low concentrations while high concentrations of the peptidomimetics in honey bee found no detectable adverse effects.

## Methods

### Chemicals

Proctolin and the amino acid replacement, alanine scan and glycine insertion analogs were synthesized by Pepmic (Jiangsu, China) and prepared for greater than 85% purity. For Chinese hamster ovarian (CHO) cell culture, DMEM/F12 medium, Fungizone® and penicillin/streptomycin were obtained from Gibco® Cell Culture at Life Technologies (Grand Island, NY). Coelenterazine h was obtained from ATT Bioquest (Sunnyvale, CA). Fetal bovine serum (FBS) was obtained from Atlas biologicals (Fort Collins, CO).

### Bioinformatics and receptor cloning

Proctolin and the receptor searches in GenBank were made by an initial query of the respective protein sequence of *Drosophila melanogaster*. When the search yielded orthology in various taxa, further expanded searches was made by using queries of closely related taxa. In the case of the proctolin receptor orthology searches, two criteria were used: back blast of the search output against the *D. melanogaster* database and clustering in the distance tree view. “Lacking” orthology was concluded after searches of the NCBI database for nr proteins, the RefSeq Genome Database, whole-genome shotgun contigs, and expressed sequence tags during December 2018.

The receptor cDNA was amplified from the total RNA isolated from a pool of 10 adult mites collected from beehives in Manhattan, Kansas^13^. Total RNA was isolated by using TRI Reagent (Zymo Research). First-strand cDNA was synthesized by using an ImProm-II™ Reverse Transcription System (Promega), and PCR amplification was performed by Q5® high-fidelity DNA polymerase (New England Biolabs). The PCR product was cloned into pGEM-T-EZ (Promega) and transferred to pcDNA3.1 + (Invitrogen) to add the *EcoR*I cloning site. The sequence was confirmed by Sanger sequencing and submitted to GenBank (Accession number MN462557.1). Phylogenetic tree was generated by using neighbor joining method with 1000 bootstrapping after alignment made by Muscle in MEGA7^51^.

### Receptor activity assay

Cells were transiently transfected with pcDNA3.1 + containing the open reading frame of *Varroa* mite proctolin receptor (VdProctR) or empty vector using TransIT®-2020 (Mirus Bio). Approximately thirty hours after transfection, the cells were collected and preincubated with coelenterazine h for the functional assay, as previously described^30,31,52^. The ligands in serial dilutions were loaded into 96-well opaque plates. Cells treated with coelenterazine h were injected into each well on an Orion microplate luminometer (Berthold) luminescent plate reader. The luminescence was measured for 20 s immediately after the cell injections. Data analyses were performed to determine the accumulated luminescence value for 20 s after extraction of blank-well luminescence. A dose–response curve was generated and the EC_50_ (50% effective concentration) was calculated in Origin 7 (OriginLab) as described in Jiang et al.^31,53^. The three biological replications were regressed for the dose–response curves with 95% confidence intervals (Table 1). For the tests of the second set of ligands, we used the cell line that stably expressed VdProctR, which were selected by Zeocin for 5 passages.

### Toxicity test

Adult female *Varroa* mites were collected using a sugar roll method from honey bee hives located at the East Campus apiary of the University of Nebraska (Lincoln, NE USA) in September–October 2019. The hives were not treated with chemicals for honey bee parasites or pathogens. The mites were sorted in the laboratory with a paint brush and tested within 4 h post collection. Only healthy mites, vigourously mobile on the filter paper, were placed in 9 cm diameter Petri dishes (Fisher Scientific) lined with filter paper. The dorsal side of the mites was secured to a strip of tape placed on the filter paper. Ten mites were secured to the tape in each dish and three dishes were used for each treatment (i.e., 30 mites/treatment). The peptide mimics were prepared for 25 mM stock solution in dimethyl sulfoxide (DMSO) and further dilutions were made in autoclaved ddH_2_O to a concentration of 0.1 nM for 2334, 2336, and Ac-2442 and 3 nM for 2326. Each peptide mimic was delivered as a 200 nL aliquot to the gnathosoma of the mites with autoclaved ddH_2_O serving as the untreated control. Following exposure to the peptide mimics, the mites were kept at high humidity for 48 h. The number of affected (i.e., dead and/paralyzed) was observed and recorded at 1, 2, 6, 12, 24, and 48 h. The percent effect for each treatment was analyzed using a one-way analysis of variance (ANOVA) with a Tukey’s post-hoc test (GraphPad Prism, La Jolla, CA; *p* < 0.05).

For injection experiments in *Varroa* mite to examine the direct physiological response to the peptidomimetics, we injected ~ 5 nL of of 200 µM compound 2334 while controls were injections of equal volume of H_2_O (n = 4 in each). Micro-glass electrodes were used for the injections using the pressure injection system (Dagan PMI-200) through anterior region of ventral idiosoma (Fig. 3F). The injections were made within 5 h after the collections of the mites as described above. Defecation induced by the injection was observed within 10 min.

For honey bee (*Apis mellifera*) assays, honey bee workers from healthy colonies were collected from Honeybee Valley of Ghent University, Belgium, and maintained in cages on sugar dough and water, under standardized conditions of 28–30 °C, 60–65% air humidity and continuous darkness^54^. The colony were maintained with a periodic sanitary control that the honeybee hives are free of pathogens, diseases and viruses. There was no *Varroa* mite infection in the honeybee hives at the moment of collecting the workers for the assays. In the preliminary/first assay, honeybee workers were injected with 10 pmol of proctolin or the proctolin analogue. This was realized by injecting a volume of 50 nL of a concentration of the compound of 200 µM. Potential effects on survival were scored at 24 h after injection. There were no lethal effects. This test was done with the following compounds were tested: 2439, 2334, 2336, Ac-2442 and controls. Subsequently, working solutions were freshly prepared prior to the bioassays by diluting the stock with distilled water to the required concentration.

In the second assay the honeybee workers were injected with 12 pmol of proctolin or the proctolin analogues. This was realized by injecting a volume of 800 nL of a concentration of the compound of 15 µM. Potential effects were scored: sublethal effects on food intake at 3 days after injection (sugar water uptake) and also survival at day 7 after injection (Table 2). This test was done the following compounds: 2330, 2331, 2334, 2335, 2336, proctolin, and controls. The controls were injected with the same amount of distilled water and a non-injected control was included as described before^55^. In brief, after injecting, each honey bee was reared individually with 50% sugar water (BioGluc, Biobest, Westerlo, Belgium) in a single housing tube, and the food intake of each honeybee was measured at 72 h after treatment (Table 2). The reduction of sugar water represented the amount of food intake by the honey bee within 3 days. Per treatment, two independent repetitions were performed, each consisting of 15 honey bees.

### Peptidomimetic analog synthesis

Analogs were synthesized on an ABI 433A peptide synthesizer with a modified FastMoc 0.25 procedure using an Fmoc-strategy starting from Rink amide resin (Novabiochem, San Diego, CA, 0.5 mM/g). The Fmoc protecting group was removed by 20% 4-methyl piperidine in DMF (Dimethyl formamide). A fourfold excess of the respective Fmoc-amino acids was activated in situ using HBTU (2-(1 h-benzotriazol-1-yl)-1,1,3,3-tetramethyluronium hexafluorophosphate) (1 eq.) /HOBt (1-hydroxybenzotriazole) (1 eq.) in NMP (N-methylpyrrolidone) or HATU (2-(7-Aza-1H-Benzotiazole-1-yl)-1,1,3,3-tetramethyluronium hexafluorophosphate) (1 eq.)/HOAt (1-hydroxy-7-azabenzotriazole) (1 eq.) in NMP for Aib and the amino acid immediately following it in the sequence. The coupling reactions were base catalyzed with DIPEA (N,N-diisopropylethylamine) (4 eq.) The amino acid side chain protecting groups were PMC for Arginine and tBu for both Threonine and Tyrosine. Acetylation was accomplished as previously described^56^.

The analogs were cleaved from the resin with side-chain deprotection by treatment with TFA (Trifluoroacetic acid):H_2_O:TIS (Triisopropylsilane) (95.5:2.5:2.5 v/v/v) for 1.5 h. The solvents were evaporated by vacuum centrifugation and the analogs were desalted on a Waters C_18_ Sep Pak cartridge (Milford, MA) in preparation for purification by HPLC. The analogs were purified on a Waters Delta-Pak C_18_ reverse-phase column (8 × 100 mm, 15 µm particle size, 100 Å pore size) with a Waters 510 HPLC system with detection at 214 nm at ambient temperature. Solvent A = 0.1% aqueous trifluoroacetic acid (TFA); Solvent B = 80% aqueous acetonitrile containing 0.1% TFA. Initial conditions were 10% B followed by a linear increase to 90% B over 40 min.; flow rate, 2 ml/min. Delta-Pak C_18_ retention times: **2393**, Ac-RYL**[Hyp]**T-OH: 3.0 min; **2336**, RYL**[Oic]**T-OH: 3.0 min; **2330**, **[dR]**YLPT-OH: 2.4 min; **2331**, RYL**[β**^**3**^**P]**T-OH: 2.8 min; **2335**, RYL**[Aib]**T-OH: 2.4 min; **2337**, R**[dY]**L**[Hyp]**T-OH: 2.5 min; **2327**, RYL**[dP]**T-OH: 2.6 min; **2328**, RY**[dL]**PT-OH: 2.6 min; **2326**, RYLP**[dT]**-OH: 2.1 min; **2329**, R**[dY]**LPT-OH: 2.4 min; **2332**, RY**[β**^**3**^**L]**PT-OH: 2.5 min; **2333**, R**[β**^**3**^**F]**LPT-OH: 2.5 min; **2334**, RYL**[Hyp]**T-OH: 2.4 min; **2340**, R**[dY]**L**[Oic]**T-OH: 2.4 min. The analogs were further purified on a Waters Protein Pak I 125 column (7.8 × 300 mm). Conditions: isocratic using 80% acetonitrile containing 0.1% TFA; flow rate, 2 ml/min. Waters Protein Pak retention times: **2393**, Ac-RYL**[Hyp]**T-OH: 7.0 min; **2336**, RYL**[Oic]**T-OH: 7.0 min; **2330**, **[dR]**YLPT-OH: 7.0 min; **2331**, RYL**[β**^**3**^**P]**T-OH: 7.0 min; **2335**, RYL**[Aib]**T-OH: 7.0 min; **2337**, R**[dY]**L**[Hyp]**T-OH: 7.5 min; **2327**, RYL**[dP]**T-OH: 7.0 min; **2328**, RY**[dL]**PT-OH: 7.5 min; **2326**, RYLP**[dT]**-OH: 7.5 min; **2329**, R**[dY]**LPT-OH: 7.0 min; **2332**, RY**[β**^**3**^**L]**PT-OH: 6.5 min; **2333**, R**[β**^**3**^**F]**LPT-OH: 6.5 min; **2334**, RYL**[Hyp]**T-OH: 7.5 min; **2340**, R**[dY]**L**[Oic]**T-OH: 7.5 min.

Amino acid analysis was carried out to quantify the analogs and to confirm identity: **2393**, Ac-RYL**[Hyp]**T-OH: L[1.0], R[1.0], T[1.0], Y[1.0]; **2336**, RYL**[Oic]**T-OH: L[1.0], R[1.0], T[1.0], Y[1.1]; **2330**, **[dR]**YLPT-OH: L[1.0], P[1.2], R[1.0], T[1.0], Y[1.1]; **2331**, RYL**[β**^**3**^**P]**T-OH: L[1.0], R[1.0], T[1.0], Y[1.1]; **2335**, RYL**[Aib]**T-OH: L[1.0], R[0.9], T[1.0], Y[1.1]; **2337**, R**[dY]**L**[Hyp]**T-OH: L[1.0], R[0.9], T[1.0], Y[1.1]; **2327**, RYL**[dP]**T-OH: L[1.0], P[1.0], R[1.0], T[1.0], Y[1.0]; **2328**, RY**[dL]**PT-OH: L[1.0], P[1.0], R[1.0], T[1.0], Y[1.0]; **2326**, RYLP**[dT]**-OH: L[1.0], P[1.0], R[1.0], T[1.0], Y[1.1]; **2329**, R**[dY]**LPT-OH: L[1.0], P[1.0], R[1.0], T[1.0], Y[1.1]; **2332**, RY**[β**^**3**^**L]**PT-OH: P[1.1], R[1.0], T[1.0], Y[1.0]; **2333**, R**[β**^**3**^**F]**LPT-OH: L[1.0], P[0.9], R[0.9], T[1.0]; **2334**, RYL**[Hyp]**T-OH: L[1.0], R[1.0], T[1.1], Y[1.0]; **2340**, R**[dY]**L**[Oic]**T-OH: L[1.0], R[1.0], T[1.1], Y[1.0]. The identity of the analogs was also confirmed by MALDI-MS on a Kratos Kompact Probe MALDI-MS instrument (Shimadzu, Columbia, Maryland). The following molecular ions (MH^+^) were observed: **2393**, Ac-RYL**[Hyp]**T-OH: 708.1 (calc. 708.0); **2336**, RYL**[Oic]**T-OH: 704.8 (calc. 703.9); **2330**, **[dR]**YLPT-OH: 649.8 (calc. 649.7); **2331**, RYL**[β**^**3**^**P]**T-OH: 663.6 (calc. 663.0); **2335**, RYL**[Aib]**T-OH: 637.6 (calc. 637.7); **2337**, R**[dY]**L**[Hyp]**T-OH: 665.6 (calc. 655.8); **2327**, RYL**[dP]**T-OH: 649.7 (calc. 649.7); **2328**, RY**[dL]**PT-OH: 649.7 (calc. 649.7); **2326**, RYLP**[dT]**-OH: 649.3 (calc. 649.0); **2329**, R**[dY]**LPT-OH: 649.7 (calc. 649.7); **2332**, RY**[β**^**3**^**L]**PT-OH: 663.5 (calc. 663.0); **2333**, R**[β**^**3**^**F]**LPT-OH: 647.4 (calc. 647.0); **2334**, RYL**[Hyp]**T-OH: 665.8 (calc. 665.7); **2340**, R**[dY]**L**[Oic]**T-OH: 703.7 (calc. 703.8).

### The molecular dynamic simulations study of the proctolin receptor docking model

The structure of proctolin receptor was built by using GPCR I-TASSER (Iterative Threading ASSEmbly Refinement) server^32–35^. The C-terminal of proctolin receptor was removed. The homology model of proctolin receptor was used to dock with protcolin. The Induced Fit Docking (IFD) method^36^ was used to obtain the preliminary results. Then the preliminary results from the IFD were used to dock by QM-Polarized Ligand Docking method. All of the docking process were implemented in Maestro software (Schrodinger Release 2019–3).

We used the previous docking model of proctolin and proctolin receptor to keep the simulation study. CHARMM-GUI was used to embed the docking model of proctolin receptor with proctolin into the 1-palmitoyl-2-oleoyl-sn-glycero-3-phosphocholine (POPC) lipid bilayer environment, and length of X and Y are 80 Å which is perpendicular to the z-direction, the length of Z is 132 Å^57^. The Cl^-^ ions were added to generate a neutral system. Amber17 was used to run the solvated structures by MD simulations and AMBER force field, ff14SB, was applied for the MD simulation protein^58^. The particle mesh Ewald method was used to calculate the long-range electrostatic interactions^59^. First, the system was minimized in 5000 steps while positional restraints for proteins, and ligand, and lipid head groups. The lipid structures were keeping dihedral restraints. Then, the system was equilibrated for 50 ps at 303.15 K (NVT ensemble) and 325 ps (NPT ensemble). The temperature was controlled by using a Langevin thermostat with a friction coefficient 1.0 ps^-1^, the pressure was controlled by Monte-Carlo barostats. The time step of the simulations was 0.002 ps, the total production time was 1.0 μs. VMD software was used to analyze the MD trajectory. Figures were processed by ChimeraX^60,61^.

## Supplementary Information

Supplementary Information.

## Data Availability

The nucleotide sequence was deposited in NCBI GenBank with the accession number MN462557.1.
