# Supplementary material for: Bee-safe peptidomimetic acaricides achieved by comparative genomics
Source: Sci Rep. 2022 Oct 14;12:17263. doi: 10.1038/s41598-022-20110-0 (PMC9568543; doi:10.1038/s41598-022-20110-0)
Supplement: Supplementary file 1 — Supplementary Information. [file 41598_2022_20110_MOESM1_ESM.docx]

**Supplementary Information**

**Proctolin sequences found in arthropods.** Yellow highlights are for the active proctolin sequence.

>XP_001964938.1 Diptera [Drosophila ananassae]

MRAARSIDQQASPGDSRLMRGGCGNGSGNGHRWLLVWMMVLLLVVPSHLVDGRYLPTRSHGDDLDKLRELMLQILELSNE

DPQTQQQQQHPLRLHNEATGNSNGNSIGSSSNINNPRVSNSNSNAAWLQKLSAMGALDELGGDAPRYGPNYGRY

>XP_017856999.1 Diptera [Drosophila arizonae]

MTLTATRNQAPSASLSSSSSPSPSATVMPSPKLQSSPSLSLSLPMPLLLSLMLLLLLLVPPQPCESRYLPTRSHGDELDK

LRELMLQILELSNEDPQQQQQQQQPHQMRLHNEANNPLTAQRGSNSGSSANAAWLQKLGAMGALDTDAGYGRY

>XP_016968639.1 Diptera [Drosophila biarmipes]

MGVTRRQGRGCGSGHRWLLVWMTVLLLAVPPHLVDGRYLPTRSHGDDLDKLRELMLQILELSNEDPQQPQQQQQQHPQLR

LHNEATSGSSNINNPRVSNGNSNAAWLQKLSAMGALDELGGDGPRFGPNYGRY

>XP_017105325.1 Diptera [Drosophila bipectinata]

MRVARSSDQGGGRLMREGCRNGSGNGNGHRWLLVWMLVLLLVVPQHLVDGRYLPTRSHGDDLDKLRELMMQILELSNEDP

QTQQQQQQQHPLRLHNEATGNSNSIGSSSSNINNPRVSNSNSNAAWLQKLSAMGALDELGGDAPRFGSNYGRY

>XP_017842664.1 Diptera [Drosophila busckii]

MNQLTYKPWGMLLLLLLLLLEPLQPCESRYLPTRSHGDELDKLREMMLQILELSNEDSQQQQQQQHQQQQLLRLHNEANN

NNNNQQQQQLTAQRGSSSNAAWLQKLGALGALDTEGVNLGNAYARY

>XP_017115861.1 Diptera [Drosophila elegans]

MSVPGSQGIGSGIGHRWLLVWMTVLLLVVPPHLVDGRYLPTRSHGDDLDKLRELMLQILELSNEDPQQQQQQQQQQQQQH

PLLRLHNEATSSNSNSNNPRVSNGNSNAAWLQKLSAMGALDELGGDGAQRFGPNYGRY

>XP_001970210.1 Diptera [Drosophila erecta]

MGVLRRQGTGIGSGSGSGHRWLLVWMTVLLLVVPPHLVDGRYLPTRSHGDDLDKLRELMLQILELSNEDPQQQQQQQQQQ

QQHPQLRLHNEAIGGSSSSSSSNSNNPRVSNGNSNAAWLQKLSAMGALDELGGDGVRFGPNYGRY

>XP_017070037.1 Diptera [Drosophila eugracilis]

MGVTRRQGSGSGHRWLLVWMTVLLLVVPPHLVDGRYLPTRSHGDDLDKLRELMLQILELSNEDPQQQQQQQHPQLRLRNE

ATSSNSNSNNNPRVSNGNSNAAWLQKLSAMGALDELGGDGVRFGPNYGRY

>XP_017060512.1 Diptera [Drosophila ficusphila]

MRAERKGQASGRGSGCGSGHRWLLVSLTVLLLVVPPHLVDGRYLPTRSHGDDLDKLRELMLQILELSNEDPQQQQQQQQH

PQLRLHNEAAGGNNNNNLGSSNNNPRVSNGNSNAAWLQKLSAMGALDELGGDGARFGPNYGRY

>XP_001989136.1 Diptera [Drosophila grimshawi]

MSVIGNRASSHKLQLSLPLAVVVVVMMLLVVVPLQPCESRYLPTRSHGDELDKLRELMLQILELSNEDPQQQQQQQQHQM

RLHNEANNPLTAQRVGNSNSNSNAAWLQKLGAMGALDTEAGYGRY

>XP_023175316.1 Diptera [Drosophila hydei]

MTQTATRNQAPSAPASLAPISSPSLTVMPSQRLQSSLSLSLPLPLLLSLMLLLLLLVPPQPCESRYLPTRSHGDELDKLR

ELMLQILELSNEDPQQQQQQQQQHQMRLHNEANNPLTAQRGSSSSNSNAAWLQKLGAIGALDTDAGYGRY

>XP_017029400.1 Diptera [Drosophila kikkawai]

MPSATPTNTTIMRGSGLGMEATQASGHRWLLVWMTVLLLVVPPHLVDGRYLPTRSHGDDLDKLRELMLQILELSNEDPQQ

QQQQQHPLRLHNEANGGSNNNNNNNLRASNGNSNAAWLQKLSAMGALDELGGDGARFAPNYGRY

>NP_609158.2 Diptera [Drosophila melanogaster]

MGVPRSHGTGIGCGSGHRWLLVWMTVLLLVVPPHLVDGRYLPTRSHGDDLDKLRELMLQILELSNEDPQQQQQQQQQQQH

PQLRLHNEATGGSSSSSNINNPRVSNGNSNAAWLQKLSAMGALDELGGDGARFGPNYGRY

>XP_002001805.1 Diptera [Drosophila mojavensis]

MTLTATRNQAPSASLSSSPSPSATAMPSPRLQSSPSLSLSLPMPLLLSLMLLLLLLVPPQPCESRYLPTRSHGDELDKLR

ELMLQILELSNEDPQQQQQQQPHQMRLHNEANNPLTAQRGSNSGSSANAAWLQKLGAMGALDTDAGYGRY

>XP_017152587.1 Diptera [Drosophila miranda]

MRAARSSQTGTERESERCLIMGMGLNLRQGHRWLVWLLLLLLAIPPQMVDGRYLPTRSHGDDLDKLRELMLQILELSNED

PQQQQQQQQQTMPQQHPLLRLHNEANSGSSSTAGGSSNGNIPRVSNGNSNAAWLQKLSAMGALDELGGDAPRMGPNYGRY

>XP_022226764.1 Diptera [Drosophila obscura]

MRAAKSSKTGTERGLNMGMGISLNLRQGHRWLVWLLLLLLAIPPQMVDGRYLPTRSHGDDLDKLRELMLQILELSNEDPQ

KQQQTMPQQHPLLRPHNEASSGSNSGTVSGSNTNTINPRVSNGNSNAAWLQKLSAMGALDELGGDAPRMGQNYGRY

>XP_002015010.1 Diptera [Drosophila persimilis]

MGMGLNLRQGHRWLVWLLLLLLAIPPQMVDGRYLPTRSHGDDLDKLRELMLQILELSNEDPQQQQQQQQQQTMPQQHPLL

RLHNEANSGSSSTAGGSSNGNNPRVSNGNSNAAWLQKLSAMGALDELGGDAPRMGPNYGRY

>XP_001356716.3 Diptera [Drosophila pseudoobscura pseudoobscura]

MRAARSSQTGTERESERCLIMGMGLNLRQGHRWLVWLLLLLLAIPPQMVDGRYLPTRSHGDDLDKLRELMLQILELSNED

PQQQQQQQQQQTMPQQHPLLRLHNEANSGSSSTAGGSSNGNNPRVSNGNSNAAWLQKLSAMGALDELGGDAPRMGPNYGR

Y

>XP_002036124.1 Diptera [Drosophila sechellia]

MGVPRRQGTEIGCGSGHRWLLVWMTVLLLVVPPHLVDGRYLPTRSHGDDLDKLRELMLQILELSNEDPQQQQQQQQQHPQ

LRLRNEATGGSSSSNINNPRVSNGNSNAAWLQKLSAMGALDELGGDGARFNPNYGRY

>XP_020799171.1 Diptera [Drosophila serrata]

MRGSVLGMGATPASGHRWLLVWMTVLLLVVPPHLVDGRYLPTRSHGDDLDKLRELMLQILELSNEDPQQQQQQQQQHPLR

LHNEANGGGGMGGIGGNNNYNLRASNGNSNAAWLQKLSAMGALDELGGDGARFAPNYGRY

>XP_016024024.1 Diptera [Drosophila simulans]

MGVPRRQGTGIGCGSGHRWLLVWMTVLLLVVPPHLVDGRYLPTRSHGDDLDKLRELMLQILELSNEDPQQQQQQQQQHPQ

LRLRNEATGGSSSSSNINNPRVSNGNSNAAWLQKLSAMGALDELGGDGARFNPNYGRY

>XP_016942496.1 Diptera [Drosophila suzukii]

MGATRRQGSGCGSGHRWLLVWMTVLLLVVPPHLVDGRYLPTRSHGDDLDKLRELMLQILELSNEDPQQQQQQQQQHPQLR

LHNEATSSNSNSNSNINNPRVSNGNSNAAWLQKLSAMGALDELGGDGPRFGPNYGRY

>XP_017013221.1 Diptera [Drosophila takahashii]

MGVTRRPGSGCGSGHRWLLVWMTVLLLVVPPHLVDGRYLPTRSHGDDLDKLRELMLQILELSNEDPQQQQPQQQQQHPQL

RLHNEATRSGSSSSGNINNPRVSNGNSNAAWLQKLSAMGALDELGGDGPRFGPNYGPY

>XP_002051865.2 Diptera [Drosophila virilis]

MTMTADRNQAQVQAQAQAEAPALATAPTLARSHRLQLSLPLPLLLALMMLLLLLVPPQPCESRYLPTRSHGDELDKLREL

MLQILELSNEDPQQQQQQSHQMRLHNEANNPLTAQRASGSSNAAWLQKLGAMGALDTEGGYGRY

>XP_002064642.2 Diptera [Drosophila willistoni]

MTMKAARNQVYGPKVLIWLLLLLLAMPPQMVDGRYLPTRSHGDDLDKLRELMLQILELSNEDPQQQQQQQQQQPLQQQQQ

HPLLRLHNEGNNPLLANSQRGSNSNAAWLQKLSAMGALDEQGGGYGRY

>XP_002087991.1 Diptera [Drosophila yakuba]

MGVPRRQGTGIGVGSGSGSGHRWLLVWMTVFLLVVPSHLVDGRYLPTRSHGDDLDKLRELMLQILELSNEDPQQQQQPQQ

QQQQQHPQLRLHNEATVGSSNSNSNSNINNPRVSNGNSNAAWLQKLSAMGALDELGGDGVRFGPNYGRY

**Other Diptera**

>XP_011207050.1 Diptera [Bactrocera dorsalis]

MVTAQLAIRRNNNNSKQHKINKKQSGTLCSSSSNGNGNGGSASAQVKCRRGGVGVASAPTLSLGLWLLPVLLVLLAWQQQ

PCEGRYLPTRSHGDDLDKLRELMLQILESSNEEQRPPNEANGNTLAQRASWLNKLNAMDGVDTQRKYGTRGIYDNGRYY

>XP_018794303.1 Diptera [Bactrocera latifrons]

MVAAQLAIRRNNANNSKQHKINKKQSGTLCSNSNGGSASAQMKCRRGGVGVASAPTLSLGLWLLPVLLVLLAWQQQPCEG

RYLPTRSHGDDLDKLRELMLQILESSNEEQRPPNEANGNTMAQRTSWLNKLNAMDGVDTQRKYGTRGIYDNGRYY

>XP_014103134.1 Diptera [Bactrocera oleae]

MVTAQLAIRRNNNKQHKSNKKQSGTLGSSSISNTSNGGSASVPVKCRRGGVGVASAPSLSLGLWLLPVLLVLLAWQQQPC

EARYLPTRSHGDDLDKLRELMLQILESSNEEQRPPNEANGNTLAQRASWLNKLNAMDGVDTQRKYGTRGIYDNGRYY

>XP_004521909.1 Diptera [Ceratitis capitata]

MVTAQLAIRRSSNANNNNNIINSNKQHKISKKQSGTMCSSSNVGSAQMKCRRKGVGVASAPSLSLGLWLLPVLLVLLAWQ

QQPCEGRYLPTRSHGDDLDKLRELMLQILESSNEEQRPPNEANGNTLAQRVSWLNKLNGLDGAEAPRKYGARGMYDNGRY

Y

>XP_023298174.1 Diptera [Lucilia cuprina]

MHFNKRGSSDNNMNLLTSSSPSYLAASTLSSSSLSSPTSSSSATKTKTTTTHGGLSLGLWWLLLMTVILMAQQQPCECRY

LPTRSHGDDLDKLRELMLQILESSNEDGGVQQRPPNEANGNTLAAAAQRANWLNKLGGNMDNMDVPRKYNSPRGIYDNGR

YY

>XP_005185657.1 Diptera [Musca domestica]

MHFNKRASNESLLTASTSSSSATELSPSALRLSSSLSSSSSAAAATTTTSPSSPAMASHAGVRLGLWCFLLLAVVLMAQQ

QPCEGRYLPTRSRGDDLDKLRELMLQILESSNEDQRPPNEANGNTMSAQRANWLNKLSGGGGSPLDGLDARKYAPHGVYD

NGRYY

>XP_013098029.1 Diptera [Stomoxys calcitrans]

MHFNKRNFNKNNSGSSESLLTAATSSSSTESSSSSSSPAAASTALRLSPLSTSTLSLSSSNSFSPLAASRGLRMGLWCFL

LLAVLLMAHQQPCEGRYLPTRSHGDDLDKLRELMLQILESSNEDQRPPNEANGNTMSAQRANWLNKLGGGGPMDGMEARK

YSAPHGVYDNGRYY

>XP_017471081.1 Diptera [Rhagoletis zephyria]

MVTAQLAIRRNSSHSSSNTNSNNRHKINNKKQSGTMCSGSGSSSSRSSTQVKCRRNGVASAPSLSLGLWLLPVLLVLLAW

QQQPCEGRYLPTRSHGDDLDKLRELMLQILESSNEEQRPPNEPNGNSLAQRASWLNKLNGVDGMDTPRKYGPRGAYDNGR

YY

>XP_011190451.1 Diptera [Zeugodacus cucurbitae]

MVTVQLAIRRNNNKQQHKINKKQSGTMCSSSSSSNGGSASAQVKCRRGGVGVASAPSLSLGLWLLPVLLVLLAWQQQPCE

GRYLPTRSHGDELDKLRELMLQILESSNEEQRPPNEANGNTLAQRASWLNKLNAMDGVETQRKYGARGMYDNGRYY

**Coleptera**

>XP_019877638.1 Coleoptera [Aethina tumida]

MFSKQLVLAMFLVMFAATFLDHVAVEARYLPTRSNGERIDKLKELLRELLENEIEKDELGDVPRWHPESRTFYKREAKPAN

>XP_018564611.1 Coleoptera [Anoplophora glabripennis]

MFSRSFVISALFVILSAILLDSQAEARYLPTRANGERVDKLRELLKELLESEIEKEEMGDVPRWHPESKLFYKREAPEVQTQPLPQQ

>AWT50618.1 Hemiptera [Diaphorina citri]

MISRKMGTFVSFTCVLLLLSCFCIGLEGRYLPTRSGNTEDRISKLKELLRDLLESEIDDYNGMASLYPRQQP

>XP_019763695.1 Coleoptera [Dendroctonus ponderosae]

MTSHAVLLMATVVVLAALVSEYRVEARYLPTRSNGDRIDKLRELLKDLLESEIDKEESETQKWRPDIKYFVKRDVEPRSNEMKSH

>XP_017777211.1 Coleoptera [Nicrophorus vespilloides]

MLLCVLAALLLINLSEVSEARYLPTRAKVDRLDKLRELLKELLESEIEKEEYQAQDAPPRWHPEQKLFYKREIPQ

>XP_022908376.1 Coleoptera [Onthophagus taurus]

MGRKMFVGVFLVMVVLFNFCGDVEARYLPTRGNGDKLDKLRELLKELLETEIENNENYDSQPRWHPESRLFYKREASAH

>XP_008190594.1 Coleoptera [Tribolium castaneum]

MFDRKLVFALVFVVFATLAVEGRYLPTRSNGDRIEKLRELLKDLFENEVEKEEYQADAPPRWHPESKLFYKREAPAH

**Hemiptera**

>XP_001949738.1 Hemiptera [Acyrthosiphon pisum]

MAGKFSALFLVGFVAAVVVAPYMMAEARYLPTRGNDDRLTRLKELLTDLLDSGAQPNLEMERPYVDVNGDFSRLRPREYN

IPEKSIMELFNPTVPHHQRPRS

>XP_015368214.1 Hemiptera [Diuraphis noxia]

MAGKFSVLFLVGFMTAVVVAPYMMAEARYLPTRGNDDRLTRLKELLNDLLDSGAQPNLEMERPYVDVNGDFSRLRSREYN

IPEKSIMELFNPTVPHHQRPRS

>AXF48201.1 Hemiptera [Laodelphax striatella]

MGLRAVLMLAVAAALVLGALITADARYLPTRRSQDDRLDRLRELLKDLLESEDHSEYDRRLFYKREMQPPLSIEQLYRQN

>XP_025195691.1 Hemiptera [Melanaphis sacchari]

MASKFSALFLVGFVAAVVVAPYIMAEARYLPTRGNEDRLTRLKELLTDLLNSGAQSNLDMERPYVDLEGDFSRLRPREYN

IPEKSIMELFNPTVPHHQRPRS

>XP_022166431.1 Hemiptera [Myzus persicae]

MASKLSVLFLVGFVAAVVVAPYMMAEARYLPTRGNDDRLTRLKELLTDLLDSGAQPNLEMERPYVDVNGDFSRLRPREYN

IPEKSIMELFNPTAPHHQRPRS

>XP_022189578.1 Hemiptera [Nilaparvata lugens]

MGLRAVLMVAVAAALILGALITVDARYLPTRRSQDDRLDRLRELLKDLLESEDHSEYDRRLFYKREMPPPLSIEQLYHQN

>AEX08669.1 Hemiptera [Rhodnius prolixus]

MATTTQSKVMSREVIVVAVLMMVLLSSSMVQSRYLPTRGADDRILRLRQLLKDLMENDLDPIMEHPAAPNGQYDPRLYKR

AAPPVQWDAVGAQFAGN

>XP_025424455.1 Hemiptera [Sipha flava]

MAVKFSGLLFVGFVATMVVVPYMMAEARYLPTRGNEDRLTRLKELLTDLLDSGVQPNLEMERPYVDSVGDYNRLRPREYS

IPEKSIMELFNPTAQHHQRPRS

>XP_026272267.1 Thysanoptera [Frankliniella occidentalis]

MRTNVAALATLMACLLGLAALTSPVAARYLPTKRGSQDGDRLDRLAHLIKELLAESDAAPMDHTAYDQRVFYKREAPSYP

ERDAAAAPLQVPDTGRAQLVAPAARN

>XP_002432200.1 Phthiraptera [Pediculus humanus corporis]

MMLRAFAVCFVILITVNFVSSRWLPTRSQEDRLDKLKEMLKDLLESEIERANIEGKYMYKRDVNTQIESIQPKNDHDDKK

LFKETNNNNKQLDTKSQL

>XP_021935204.1 Blattodea; Isoptera [Zootermopsis nevadensis]

MCCRQAVLLALMLVVMYAATEARYLPTRSQDDRLDRLRELLRDLLESEVEKTNVNNNSYDRRMLYKRQVPMITTEQQQAPLVSAQQ

>XP_021949823.1 Collembola [Folsomia candida]

MAKIMNQCSVRMMITLVIAAFLTSQVLVTVSARYLPTRSQDDRLEKLRELLRDLLEGDLERRPATSGVDYEMMAQQGRPPVYKRSTNAYTDLDAAAAAGKVGPNFLHRIVPNLRKSLD

>ODN05925.1 Collembola [Orchesella cincta]

MSGGGGRSATGNINGQCAWFLLLISSAEREILNTKTLKRMEQLSVRVALVLLAAAFLASTSTMVAVSARYLPTRSQEDRL

ERLRELLKDLFDSDYDHRTYPLADVSVLPERAAIFKRSAGLAYAPGVTGHDLVKGLRNGALSSSAALGGNGLVQLPSAML

DSPYADPERKLI

>XP_018020603.1 Crustacea [Hyalella azteca]

MKGSLKLVVALAVLVVVLGMLGVCEGRYLPTRGDEARLEEIRMMLRDLLEGAAGGAPRLVKRDVETDNFQARNSLT

>AWK57540.1 Crustacea [Cherax quadricarinatus]

MARSGMLVVMALVVLVAALTNARYLPTRADDSRLEEIRELLREVLERTADGGSSISSSSSRVSGSGYDKRFLFKRAAAAE

GGVAGEVVEPLLNLPQ

>LAA00479.1 Arachnida [Parasteatoda tepidariorum]

MSVIRKEVMALTLLTLLSCSWIASGRYLPTRSDSTRREQIKELLGALLDLAPEDRDIRRSSYPYDLHSGS

MAKRSVQEPERSIPFQRDAE

>XP_023234384.1 Arachnida [Centruroides sculpturatus]

MRASQMVVMCVVILVAVSCTVVQGRYLPTRSDDARREQIKEILRALLELTPEDRLFARMGYAPYDYREEGHLTKRSVGGS

VDSYRHDDDQMAY

>KFM66071.1 Arachnida [Stegodyphus mimosarum]

MSAVKHSLVCIFLILLVCATLRVDGRYLPTRSDSSRYERIKEVLRALLELNDAEFQNSRQNFAYDDVMRSKRDASNQHPS

SPVHV

>XP_025016906.1 Arachnida [Tetranychus urticae]

MLNFGSKQYHHILLTMVVVVIVSTIVSARYLPTRNDDTRKEQIKELLRMFLEASNSSPDVDSIQGTAKRSPGSLFDYRGT

NNPSYLIKRTAIEEVPSGDLAA

>Arachnida Varroa destructor

MSSPMSARGILWGLILVGVILLSLSVESQARYLPTRADPARRERIREILRALLLLSPGEPEARGPYTSSYEYGTGAGDLKGLERSDWDSSST*

>Arachnida Ixodes scapularis

MMVSQTRLLALAVMSTLMLLVVDARYLPTRSDDLQKDHIRDILRGLFEKAEFEKSASNLLADLGSAYTLRGGAPLGSDMGAYAGSRAGAVRSGLLSRDMGA

**Blattodea sequences partially matching in the C-terminal region of the *Zootermopsis nevadensis proctolin***

Periplaneta americana strain Shanghai urban area PaSCF18558, whole genome shotgun sequence. Sequence ID: [PGRX01003422.1](https://www.ncbi.nlm.nih.gov/nuccore/PGRX01003422)Length: 300293Number of Matches: 1

Query 47 SEVEKTNVNNNSYDRRMLYKRQVPMITTEQQQAPLVSAQQ 86

SE+EK+NVNN Y+RRMLYKR+VPMI TEQQQ PLVSAQQ

Sbjct 202192 SEIEKSNVNN--YERRMLYKREVPMIATEQQQLPLVSAQQ 202079

Cryptotermes secundus scaffold614, whole genome shotgun sequence

Sequence ID: [NEVH01010480.1](https://www.ncbi.nlm.nih.gov/nuccore/NEVH01010480)Length: 2306319Number of Matches: 1

Query 47 SEVEKTNVNNNSYDRRMLYKRQVPMITTEQQQAPLVSAQQ 86

SE EKTN NS++RR+LYKR+VPM+ TEQQQAPLV+A+Q

Sbjct 413533 SEAEKTN--GNSFERRILYKREVPMVGTEQQQAPLVAAEQ 413420

Blattella germanica strain American Cyanamid = Orlando Normal contig_23379, whole genome shotgun sequence

Query 47 SEVEKTNVNNNSYDRRMLYKRQVPMITTEQQQ-APLVSAQQ 86

SE+EKTNVNN Y+RRM+YKR+VPM+ +EQQQ APL++AQQ

Sbjct 23131 SEIEKTNVNN--YERRMIYKREVPMMASEQQQHAPLMAAQQ 23015
